# Cranberry Polyphenols in Esophageal Cancer Inhibition: New Insights

**DOI:** 10.3390/nu14050969

**Published:** 2022-02-24

**Authors:** Katherine M. Weh, Yun Zhang, Connor L. Howard, Amy B. Howell, Jennifer L. Clarke, Laura A. Kresty

**Affiliations:** 1 Rogel Cancer Center and Department of Surgery, Thoracic Surgery Section, University of Michigan, Ann Arbor, MI 48109, USA; kweh@med.umich.edu (K.M.W.); yunzh@med.umich.edu (Y.Z.); conhow@med.umich.edu (C.L.H.); 2 Marucci Center for Blueberry and Cranberry Research, Rutgers University, Chatsworth, NJ 08019, USA; ahowell@njaes.rutgers.edu; 3 Departments of Statistics and Food Science and Technology, University of Nebraska-Lincoln, Lincoln, NE 68588, USA; jclarke3@unl.edu

**Keywords:** Barrett’s esophagus, esophageal adenocarcinoma, cranberry polyphenols, proanthocyanidins, anthocyanins, flavonoids, glycosides, reverse phase protein array

## Abstract

Esophageal adenocarcinoma (EAC) is a cancer characterized by rapidly rising incidence and poor survival, resulting in the need for new prevention and treatment options. We utilized two cranberry polyphenol extracts, one proanthocyanidin enriched (C-PAC) and a combination of anthocyanins, flavonoids, and glycosides (AFG) to assess inhibitory mechanisms utilizing premalignant Barrett’s esophagus (BE) and EAC derived cell lines. We employed reverse phase protein arrays (RPPA) and Western blots to examine cancer-associated pathways and specific signaling cascades modulated by C-PAC or AFG. Viability results show that C-PAC is more potent than AFG at inducing cell death in BE and EAC cell lines. Based on the RPPA results, C-PAC significantly modulated 37 and 69 proteins in JH-EsoAd1 (JHAD1) and OE19 EAC cells, respectively. AFG treatment significantly altered 49 proteins in both JHAD1 and OE19 cells. Bioinformatic analysis of RPPA results revealed many previously unidentified pathways as modulated by cranberry polyphenols including NOTCH signaling, immune response, and epithelial to mesenchymal transition. Collectively, these results provide new insight regarding mechanisms by which cranberry polyphenols exert cancer inhibitory effects targeting EAC, with implications for potential use of cranberry constituents as cancer preventive agents.

## 1. Introduction

In recent years, rates of esophageal adenocarcinoma (EAC) and the only known precursor lesion, Barrett’s esophagus (BE), have increased sharply in the United States and the Westernized world. Mechanisms by which BE progresses to EAC are still being unraveled, but repeated exposure of the esophagus to bile at an acidic pH can result in gastroesophageal reflux disease (GERD), the primary risk factor for EAC. Subsequent inflammation, loss of barrier integrity and molecular alterations resulting from GERD contribute to EAC progression [1,2]. *TP53* mutation is recognized as the strongest known driver for EAC progression, documented to occur in over 70% of EAC cases [3,4]. Overall, mutational burden for EAC is considered high, but mutational events beyond *TP53* tend to be spread across a large number of genes (i.e., *ARID1A*, *CDKN2A*, *SMAD4*, *CTNNB1*, *EGFR*, *ERBB2*, *MET*) at relatively low frequency, as recently reviewed [5]. EAC disproportionality occurs in males and is obesity linked [6]. Efficacious prevention and treatment strategies are urgently needed considering that EAC is the seventh leading cause of cancer mortality among Caucasian males in the United States and one with a 5-year survival rate consistently below 20% [7].

Current research shows that cranberry proanthocyanidins (C-PAC) have a role in maintaining urinary tract health [8,9,10], and recently a number of in vitro investigations have reported that cranberry extracts impact multiple cancer-associated processes in 52 cancer cell lines including those derived from breast, colon, esophagus, lung, oral cavity and prostate [11,12,13,14,15]. To date, there have been twelve in vivo reports assessing the efficacy of cranberry products as cancer inhibitors; of these, six were xenograft studies, five were chemical carcinogen-induced bioassays and one was in a genetically modified mouse colon cancer model [11,16,17,18]. Our laboratory has previously shown that C-PAC treatment alters 5 miRNAs linked to *TP53*, angiogenesis, T-cell activation and apoptosis in three human EAC cell lines [19]. Furthermore, C-PAC altered the phase of cell cycle and induced caspase independent cell death [20]. The primary form of cell death induced by C-PAC in acid-sensitive EAC cell lines is autophagy, whereas acid-resistant EAC cell death occurs via cellular necrosis [20]. Furthermore, plant polyphenols are documented to modulate reactive oxygen species (ROS) in cell culture, which in turn influences signaling mechanisms resulting in either cell survival or death [21]. C-PAC induced ROS in JHAD1 and OE19 cells and interestingly, decreased ROS in CP-C BE cells [22], both resulting in cell death.

The current study sought to investigate cancer-related proteins and signaling networks associated with cranberry polyphenol treatment utilizing the reverse phase protein array (RPPA) platform which simultaneously examines the levels of 304 proteins using validated antibodies. First, we performed viability assays in BE and EAC cell lines to determine appropriate concentrations of the two cranberry polyphenol fractions, including a proanthocyanidin rich fraction (C-PAC) and a second fraction comprised of anthocyanins, flavonoids and glycosides (AFG). Next, we assessed protein modulation using RPPA technology applied to lysates collected 24 h following treatment with C-PAC or AFG in JH-EsoAd1 (JHAD1) and OE19 EAC cell lines. We performed bioinformatic analysis to investigate the process networks and pathway maps significantly modulated by C-PAC or AFG in human EAC cell lines. In addition, we validated our results by assessing levels of 18 proteins by Western blot analysis. Multiple proteins related to P53, inflammation, metastasis, DNA damage, cell cycle, cell growth arrest and NOTCH signaling were dysregulated in BE and EAC cells with mitigation by C-PAC or AFG treatment. These results are consistent to those previously observed for C-PAC treatment in EAC cells but extend mechanistic insights through identification of additional signaling mechanisms by which C-PAC or AFG exert inhibitory effects in BE and EAC cell lines. In summary, cranberry polyphenols may represent a viable prevention option for targeting EAC, a cancer characterized by high mortality due to late stage diagnosis coupled with a lack of efficacious treatment options.

## 2. Materials and Methods

### 2.1. Cranberry Polyphenol Preparation

Cranberry polyphenols were prepared as previously described with minor modifications [20]. Briefly, cranberry fruit (*Vaccinium macrocarpon* Ait.) of the “Early Black” cultivar were collected at the Marucci Center for Blueberry and Cranberry Research in Chatsworth, NJ, USA. C-PAC and AFG were isolated from cranberries utilizing solid-phase chromatography according to well established previously published methodology [8,23,24]. Following homogenization of cranberries in 70% aqueous acetone, the resulting mixture was filtered and the pulp discarded. The extract was subsequently dissolved in ethanol and separated using a Sephadex LK-20 column (600 mm × 50 mm, Sigma Aldrich, St. Louis, MO, USA). Collected fractions were monitored by thin-layer chromatography and pooled into C-PAC or AFG fractions. The absence of absorption at 360 nm and 450 nm confirmed that all but proanthocyanidins were removed from C-PAC fraction. Additional methods including 13^C^ NMR, electrospray mass spectrometry, matrix-assisted laser desorption/ionization time-of-flight mass spectrometry and acid catalyzed degradation with phloroglucinol were utilized to further verify the presence of A-type linkages, as well as to determine the concentration of proanthocyanidins in the purified extract. Anthocyanins, flavonoids and glycosides were analyzed according to Brown and Shipley [25,26] and as previously described. Purified freeze-dried C-PAC or AFG was stored at −80 °C until weighed out for experiments.

### 2.2. Cell Lines and Dose Determination Using Calcein-AM Based Viability Assay

Authenticated BE cell lines, CP-B (isolated from a “progressor” or patient who progressed to EAC) and CP-C (“non-progressor”) and EAC cell lines, JHAD1 and OE19, were utilized in this series of experiments. BE cell lines are h-TERT immortalized cell lines originally isolated from patients in the Seattle Barrett’s Esophagus study [27] and are available from American Type Culture Collection (ATCC) as CRL-4028 (CP-B) and CRL-4029 (CP-C). BE cells were cultured in BE growth medium with 5% fetal bovine serum (FBS) as recommended by ATCC. JHAD1 cells were isolated from a distal EAC, stage III, N0 in 1997 (kind gift from Dr. James Eshleman, John Hopkins University, Baltimore, MD, USA) [28] and OE19 cells were isolated in 1993 from an adenocarcinoma at the gastro–esophageal junction, stage III, N1 (ECACC, Wiltshire, UK). EAC cells were cultured in RPMI medium containing 2 mM L-glutamine, 10^4^ units/mL penicillin, 10^4^ µg/mL streptomycin, 1 mM sodium pyruvate and 10% FBS as previously reported [20,22,29]. All cell culture reagents were from Thermo Fisher Scientific (Waltham, MA, USA) or Sigma Aldrich (St. Louis, MO, USA). Cells were maintained as monolayers at 37 °C with 95% O_2_ and 5% CO_2_. In all tissue culture assays, FBS was used at a final concentration of 5% and the vehicle was 0.001% ethanol diluted in the appropriate medium. All cell lines used in these experiments were used at a passage of 9 and below.

For viability assays, CP-B (8 × 10^3^ cells/well), CP-C (8 × 10^3^ cells/well), JHAD1 (9 × 10^3^ cells/well) and OE19 (13 × 10^3^ cells/well) cells were plated into black-walled clear bottom 96-well plates and allowed to adhere overnight prior to treatment with C-PAC (75–150 µg/mL) or AFG (200–600 µg/mL). C-PAC concentrations were based on our previously published research [20,29] and AFG concentrations were optimized herein. Cell viability was determined using the Calcein AM LIVE/Dead Viability/Cytotoxicity kit for Mammalian cells from Life Technologies (ThermoFisher Scientific, Waltham, MA, USA) per the manufacturer’s instructions at 24, 48 and 72 h post-treatment. Fluorescence imaging for the viability assay was conducted using the SpectraMax^®^ MiniMax™ Imaging Cytometer (Molecular Devices, San Jose, CA, USA) with excitation/emission wavelengths of 460/535 nm.

### 2.3. Lysate Collection and Western Blot Analysis of BE and EAC Cells Treated with Cranberry Polyphenols

CP-B (2 × 10^6^ cells/T25), CP-C (2 × 10^6^ cells/T25), JHAD1 (2.25 × 10^6^ cells/T75) and OE19 (4 × 10^6^ cells/T75) cells were seeded in T25 or T75 flasks (Corning, ThermoFisher Scientific, Waltham, MA, USA) and allowed to adhere overnight prior to treatment with C-PAC (75 µg/mL) or AFG (400 µg/mL). Cell lysates were harvested at 24 h post-treatment using RPPA lysis buffer (1% Triton X-100, 50 mM HEPES, pH 7.4, 150 mM NaCl, 1.5 mM MgCl_2_, 1 mM EGTA, 100 mM NaF, 10 mM sodium pyrophosphate, 1 mM sodium orthovanadate, 10% glycerol) with complete EDTA-free protease and PhosSTOP phosphatase inhibitors (Sigma Aldrich, St. Louis, MO, USA). Protein was quantified using the DC protein assay (Bio-Rad, Hercules, CA, USA) and 15 µg/lane loaded in precast 4–20% Criterion TGX gels (Bio-Rad). Immunoblotting was performed using commercially available antibodies from Abcam (Cambridge, MA, USA): COX-2 (ab15191; 1:1000), MMP-9 (ab76003; 1:1000) and NRF2 (ab62352; 1:400); Cell Signaling Technology (Danvers, MA, USA): BCL-2 (#2876; 1:1000), GAPDH (#2118; 1:20,000), L1CAM (#89861; 1:1000), MCL-1 (#5453; 1:1000), NF-κB1 (#13586; 1:400), NOTCH1 (#3268; 1:1000), NOTCH2 (#5732; 1:1000), PARP (#9532; 1:1000), Phospho-AKT^Ser473^ (#4060; 1:1000), Phospho-AKT^Thr308^ (#13038; 1:1000), Phospho-H2AX^Ser139^ (#2577; 1:1000), Phospho-RPS6^Ser235/Ser236^ (#2211; 1:1000) and TIGAR (#14751; 1:1000); Millipore Sigma (Burlington, MA, USA): P53 (#OP-43; 1:1000); and Santa Cruz Biotechnology (Dallas, TX, USA): Cyclin B1 (sc-7393; 1:1000), GAPDH (sc-32233; 1:20,000), HSP60 (sc-13966; 1:5000) and PCNA (sc-7907; 1:200). Expression values were determined by chemiluminescent immunodetection and analyzed using ImageLab software (Bio-Rad, Hercules, CA, USA) with normalization to the loading control protein. Fold-change from vehicle was calculated.

### 2.4. Functional Proteomics by RPPA

JHAD1 and OE19 cells were treated for 24 h with vehicle, C-PAC (75 µg/mL) or AFG (400 µg/mL), and lysates were collected in duplicate and diluted to a final concentration of 1 µg/uL with 4X SDS sample buffer (40% glycerol, 8% SDS, 0.25M Tris-HCl, pH 6.8, 10% beta-mercapto-ethanol), boiled for 5 min and stored at −80 °C until processed at the MD Anderson Cancer Center RPPA facility. Five serial 2-fold dilutions of each sample were arrayed onto nitrocellulose-coated slides, probed with antibodies by tyramide-based signal amplification and visualized by DAB colorimetric reaction. Slides were scanned and the density was quantified using an Array-Pro analyzer (Media Cybernetics L.P., Silver Spring, MD, USA). Relative protein levels for each sample were determined by interpolation of each dilution curve from the standard curve of the slide. All data points were normalized for protein loading and transformed to linear values. Antibody staining was assessed through a quality control (QC) score and only data with a QC score greater than 0.8 was included in the analysis. The antibodies used by the RPPA Facility can be found at https://www.mdanderson.org/research/research-resources/core-facilities/functional-proteomics-rppa-core/antibody-information-and-protocols.html (accessed on 30 November 2021), with list 304 specifically used in our studies.

### 2.5. Bioinformatic Analysis of RPPA Data

Two-tail Student’s *t*-tests were used to determine significantly altered proteins (*p* < 0.05) using R (version 3.6.2, R Core Team, https://www.R-project.org, accessed on 23 July 2021). Mean-centered Log_2_ expression values of significant markers were used for hierarchical clustering and heatmaps generated in R using the ComplexHeatmap package (version 2.6.2, https://www.bioconductor.org/packages/release/bioc/html/ComplexHeatmap.html, accessed on 23 July 2021). Cluster lists containing statistically significant proteins (*p* ≤ 0.05) based on treatment were matched to gene names, Entrez gene IDs, Log_2_ fold change values and two-tailed Student’s *t*-test values for each RPPA marker. All statistically significant cluster lists for each comparison were uploaded and analyzed in Metacore™ and Cortellis Solution software (Clarivate Analytics, London, UK, https://clarivate.com/products/metacore/, accessed on 9 November 2021) using the default setting for marker mapping to Entrez gene IDs, followed by pathway and gene ontology (GO) enrichment analyses. Specifically, we evaluated “Pathway Maps” and “Process Networks”. *p* and FDR values for each result were calculated by using the default Metacore^TM^ database (https://portal.genego.com/, accessed on 9 November 2021) for Homo sapiens as the background and significance indicated by *p* and FDR < 0.05. Use of the default setting allows for inclusion of additional proteins found in groups or complexes in addition to the significantly altered proteins identified by RPPA. Additionally, we performed enrichment analysis using the “Avoid any protein groups/complexes” setting for marker mapping to Entrez gene IDs to confirm result findings. Results from our latter secondary analysis paralleled those obtained under the default setting. All process networks and pathway maps presented using the default analysis setting identified in Section 3.2. remained significant with *p* and FDR levels < 0.05 when analyzed using the “avoid complexes” setting, which generated all presented results herein.

### 2.6. Statistical Analyses

Prism (version 9.3.1, GraphPad Software, San Diego, CA, USA) was used for evaluating statistical differences by treatment. Viability data were evaluated for statistical significance using One-way ANOVA with Tukey’s post-hoc test where multiple conditions were assessed. *p*-values < 0.05 were considered significant. RPPA data were analyzed as described above.

## 3. Results

### 3.1. Cranberry Polyphenols Inhibit Cell Viability of Premalignant Barrett’s Esophageal and Adenocarcinoma Derived Cell Lines

**Figure 1:**
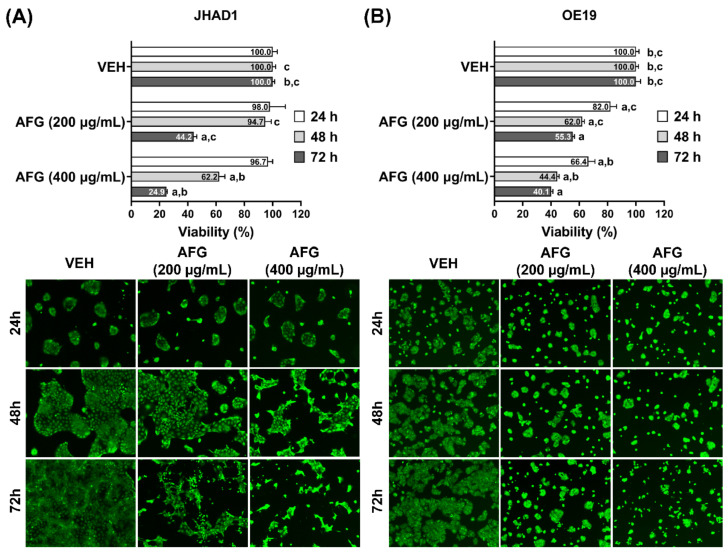
AFG induces cell death in EAC cell lines. Viability of (**A**) JHAD1 and (**B**) OE19 cells was assessed using the live stain Calcein-AM following treatment with AFG (200 and 400 µg/mL). The data are reported as the mean ± standard error of the mean (SEM) of at least four wells per treatment. Each panel contains representative fluorescent images of viable JHAD1 and OE19 cells at each time point assessed. Viability was analyzed by One-way ANOVA with Tukey’s post-hoc test (*p* < 0.05) where multiple conditions were assessed. Treatments were significantly different from a = vehicle, b = AFG (200 µg/mL) and c = AFG (400 µg/mL). AFG, fraction comprised of anthocyanins, flavonoids and glycosides; VEH, vehicle; EAC, esophageal adenocarcinoma; JHAD1, JH-EsoAd1; OE19, OE19 esophageal adenocarcinoma cell line; h, hours.

Our group had previously determined the sensitivity of EAC cell lines to C-PAC treatment, with the LD_50_ for both JHAD1 and OE19 cell lines determined to be 50 to 75 µg/mL [29]. We performed fluorescence based Calcein-AM viability assays at 24, 48 and 72 h post treatment using 200 and 400 µg/mL AFG (Figure 1) in JHAD1 and OE19 cell lines. AFG significantly inhibits the viability of JHAD1 and OE19 cells in a time and dose dependent manner. However, JHAD1 cells appear more resistant to AFG treatment at both 24 h and 48 h compared to OE19 cells. A greater magnitude of AFG-induced cell death is evident in OE19 cells compared to JHAD1 cells at both dose levels at 24 and 48 h. Conversely, JHAD1 cells are significantly inhibited by AFG at 72 h, exceeding levels of death induction noted in OE19 cells at this time point, suggesting delayed sensitivity, as shown in Figure 1A.

At 48 h post treatment, OE19 cells are 1.6-fold more sensitive to AFG-induced cell death compared with JHAD1 cells (Figure 1B). However, at 72 h 400 µg/mL AFG inhibits OE19 viability by 60% compared with 75% inhibition of JHAD1 cells. The latter may support less cytotoxicity and stronger anti-proliferative effects, resulting in cell cycle dysregulation following AFG treatment of JHAD1 cells relative to treatment effects in OE19 cells.

**Figure 2:**
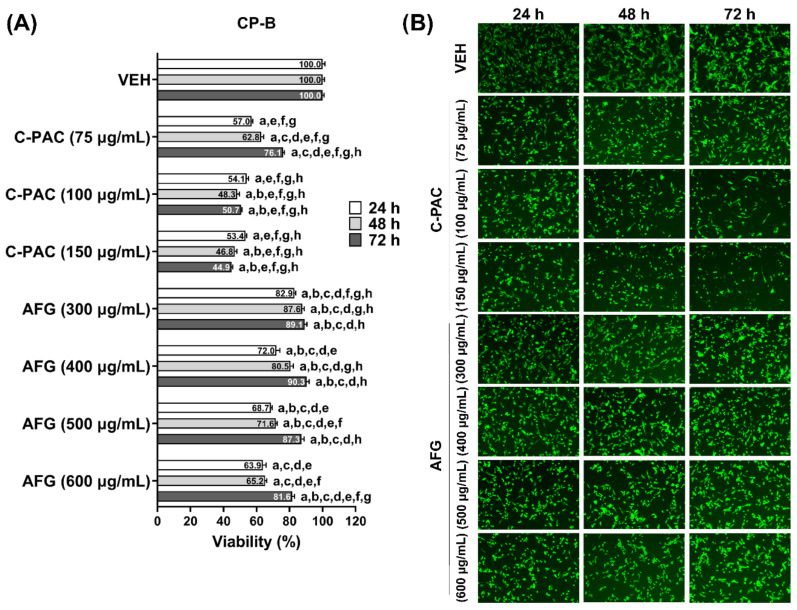
Cranberry polyphenols induce cell death in progressor BE cell line CP-B. (**A**)Viability of CP-B cells was assessed using the following treatment with C-PAC (75, 100 and 150 µg/mL) or AFG (300, 400, 500 and 600 µg/mL). The data are reported as the mean ± SEM of at least three wells per treatment. (**B**) Representative fluorescent images of viable CP-B cells at each time point assessed. Viability was analyzed by One-way ANOVA with Tukey’s post-hoc test (*p* < 0.05) where multiple conditions were assessed. Treatments were significantly different from a = vehicle, b = C-PAC (75 µg/mL), c = C-PAC (100 µg/mL), d = C-PAC (150 µg/mL), e = AFG (300 µg/mL), f = AFG (400 µg/mL), g = AFG (500 µg/mL) and h = AFG (600 µg/mL). C-PAC, proanthocyanidin rich fraction; BE, Barrett’s esophagus; CP-B, isolated from a “progressor” or patient who progressed to esophageal adenocarcinoma.

**Figure 3:**
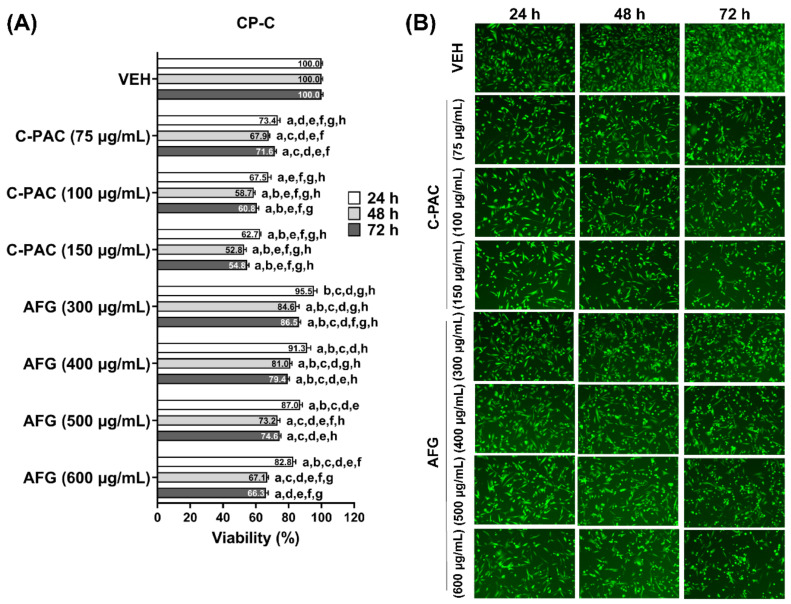
Cranberry polyphenols induce cell death in non-progressor BE cell line CP-C. (**A**) Viability of CP-C cells was assessed following 24 h treatment with C-PAC (75, 100 and 150 µg/mL) or AFG (300, 400, 500 and 600 µg/mL). The data are reported as the mean ± SEM of at least three wells per treatment. (**B**) Representative fluorescent images of viable CP-C cells at each time point assessed. Viability was analyzed by One-way ANOVA with Tukey’s post-hoc test (*p* < 0.05) where multiple conditions were assessed. Treatments were significantly different from a = vehicle, b = C-PAC (75 µg/mL), c = C-PAC (100 µg/mL), d = C-PAC (150 µg/mL), e = AFG (300 µg/mL), f = AFG (400 µg/mL), g = AFG (500 µg/mL) and h = AFG (600 µg/mL).

Additional studies determined the sensitivity of premalignant BE cells to C-PAC and AFG-induced cell death. We determined that CP-B (Figure 2) and CP-C (Figure 3) cells are more sensitive to C-PAC induced cell death compared to induction by AFG. C-PAC significantly inhibits the viability of BE cell lines in a time and dose dependent manner with the LD_50_ in the 100 to 150 µg/mL range. Thus, BE derived cell lines require a higher concentration of C-PAC to inhibit cell viability than do EAC cells where the LD_50_ is in the 50 to 75 µg/mL range. Similarly, BE cells were more resistant to AFG-induced cell death. The LD_50_ for AFG in EAC cells is evident at 200 µg/mL, yet not reached at 600 µg/mL in CP-B or CP-C BE cells (Figure 2 and Figure 3). Representative fluorescent stained images from each cell line further illustrate decreases in viability over time for CP-B and CP-C cells treated with C-PAC and AFG (Figure 2B and Figure 3B).

### 3.2. RPPA Analysis of Lysates from Esophageal Adenocarcinoma Cells Treated with C-PAC or AFG Reveal Modulation of Numerous Cancer-Linked Processes

To better understand the mechanisms by which C-PAC or AFG inhibit viability of JHAD1 and OE19 EAC cells, we utilized RPPA technology to simultaneously interrogate 304 antibodies to proteins, many implicated in cancer [30,31,32]. JHAD1 and OE19 cells were treated with either C-PAC (75 µg/mL) or AFG (400 µg/mL) for 24 h and collected lysates probed with validated antibodies. Significantly (*p* ≤ 0.05) modulated proteins were analyzed in Metacore to investigate enriched pathway and process networks linked to protein level changes modulated by C-PAC or AFG.

**Figure 4:**
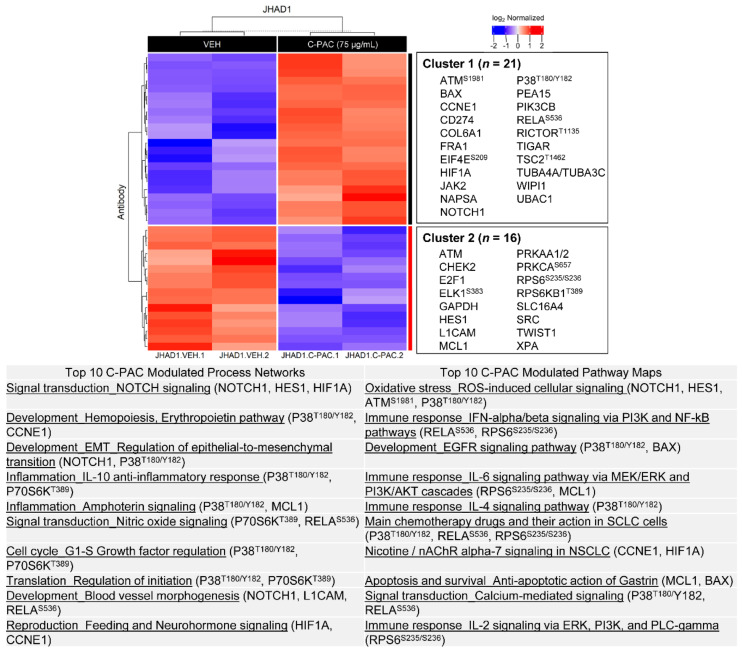
Cranberry proanthocyanidins modulate multiple process networks and pathway maps in JHAD1 cells. All significantly upregulated (red; *n* = 21) and downregulated (blue; *n* = 16) proteins from RPPA in JHAD1 cells were used to generate heatmaps following C-PAC (75 µg/mL) treatment for 24 h (*p* < 0.05). Upregulated and downregulated proteins are represented in Cluster 1 and 2, respectively. Below the heatmap is a table containing the top 10 modulated process networks and pathway maps for all proteins significantly changed with C-PAC treatment. Representative proteins in each process network or pathway map are listed. RPPA, reverse phase protein array.

**Figure 5:**
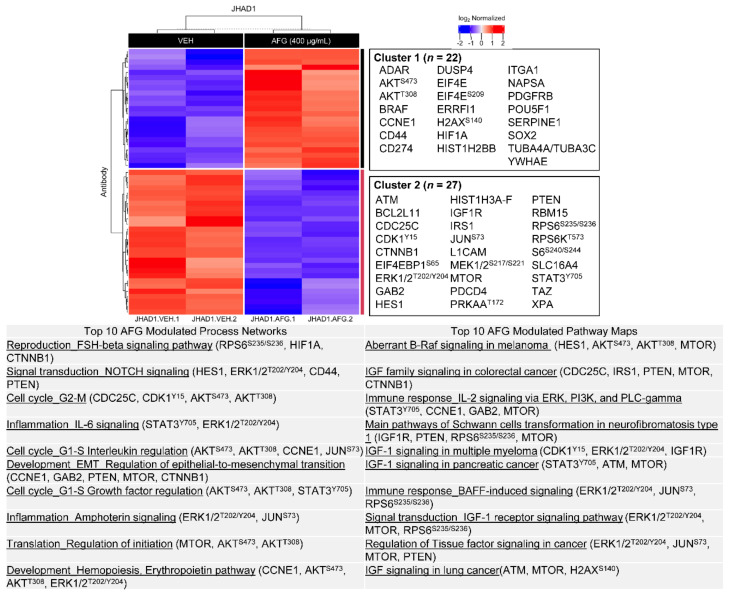
AFG modulates multiple process networks and pathway maps in JHAD1 cells. All significantly upregulated (red; *n* = 22) and downregulated (blue; *n* = 27) proteins from RPPA in JHAD1 cells were used to generate heatmaps following AFG (400 µg/mL) treatment for 24 h (*p* < 0.05). Upregulated and downregulated proteins are represented in Cluster 1 and 2, respectively. Below the heatmap is a table containing the top 10 modulated process networks and pathway maps for all proteins significantly changed with AFG treatment. Representative proteins in each process network or pathway map are listed.

The enrichment analysis results for significantly altered proteins in JHAD1 cells are presented in Figure 4 and Figure 5 for C-PAC and AFG treatment, respectively. C-PAC treatment resulted in significant modulation of 37 proteins, 21 upregulated and 16 downregulated as listed to the right of the heatmaps (Figure 4). C-PAC significantly modulated process networks including those related to signal transduction via NOTCH and nitric oxide signaling, regulation of epithelial to mesenchymal transition (EMT), anti-inflammatory pathways involving IL-10, inflammatory pathways associated with amphotericin, regulation of translation initiation and G1-S cell cycle networks. Process networks were driven by significant changes in NOTCH1, HES1, HIF1A, CCNE1, MCL1, RELA and phosphorylation of P38 at T180/Y182 (Figure 4; Supplementary Materials Table S1). C-PAC modulated pathway maps including those related to reactive oxygen species (ROS) signaling, immune response signaling via IFN- α/β, IL-2, IL-4 and IL-6, and epidermal growth factor receptor (EGFR) signaling implicating BAX and NF-κB. Additional pathways identified included apoptosis and calcium-mediated signaling. Similar to modulated process networks, proteins driving identified pathway maps included NOTCH1, HES1, and phosphorylation of P38 at T180/Y182 and RPS6 at S235/S236.

With respect to AFG treatment, 49 proteins were significantly modulated in JHAD1 cells, with 22 upregulated and 27 downregulated. As detailed in Figure 5 and Supplementary Materials Table S2, AFG significantly modulates process networks including signal transduction by NOTCH signaling, cell cycle (G1-S), inflammatory signaling involving IL-6 and amphotericin, regulation of EMT and regulation of translation initiation, with similarity to C-PAC-induced changes. The majority of altered process networks were driven by significant changes in MTOR, phosphorylation of AKT at S473 and T308, ERK1/2 at T202/Y204 and RPS6 at S235/S236. AFG treatment also modulated levels of CCNE1, HES1, HIF1A and CTNNB1. In turn, top pathway maps significantly dysregulated by AFG included those related to insulin growth factor (IGF) signaling and immune signaling through IL-2 and BAFF (tumor necrosis factor superfamily member 13b). Similarly, the proteins implicated in these pathways included HES1, MTOR, PTEN and phosphorylation of AKT at S473 and T308, RPS6 at S235/S236, ERK1/2 at T202/Y204 and H2AX at S140.

**Figure 6:**
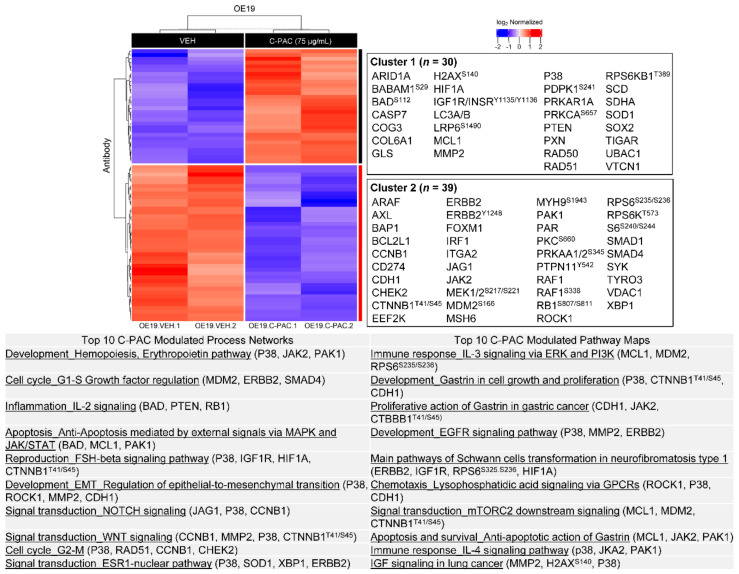
Cranberry proanthocyanidins modulate multiple process networks and pathway maps in OE19 cells. All significantly upregulated (red; *n* = 30) and downregulated (blue; *n* = 39) proteins from RPPA in OE19 cells were used to generate heatmaps following C-PAC (75 µg/mL) treatment for 24 h (*p* < 0.05). Upregulated and downregulated proteins are represented in Cluster 1 and 2, respectively. Below the heatmap is a table containing the top 10 modulated process networks and pathway maps for all proteins significantly changed with C-PAC treatment. Representative proteins in each process network or pathway map are listed.

**Figure 7:**
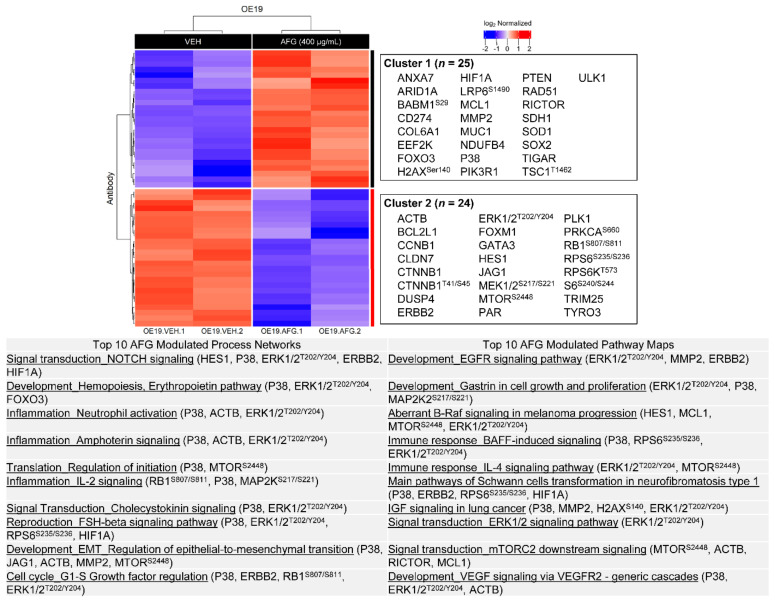
AFG modulates multiple process networks and pathway maps in OE19 cells. All significantly upregulated (red; *n* = 25) and downregulated (blue; *n* = 24) proteins from RPPA in OE19 cells were used to generate heatmaps following AFG (400 µg/mL) treatment for 24 h (*p* < 0.05). Upregulated and downregulated proteins are represented in Cluster 1 and 2, respectively. Below the heatmap is a table containing the top 10 modulated process networks and pathway maps for all proteins significantly changed with AFG treatment. Representative proteins in each process network or pathway map are listed.

Figure 6 and Figure 7 present the enrichment analysis results for significantly altered proteins in OE19 cells following C-PAC and AFG treatment, respectively. A total of 69 proteins are significantly modulated by C-PAC treatment, 30 upregulated and 39 downregulated as shown adjacent to the heatmap in Figure 6. Significantly modulated process networks include those related to cell cycle (G1-S and G2-M), IL-2 inflammatory signaling, anti-apoptosis signaling, regulation of EMT and signaling through NOTCH and WNT. Top process networks identified are driven by significant changes in P38, JAK2, PAK1, BAD, HIF1A, CTNNB1, MCL1, MDM2, MMP2 and ERBB2 (Figure 6; Supplementary Materials Table S3). C-PAC significantly modulated pathway maps including those related to IL-3 and IL-4 immune signaling, gastrin-linked cell proliferation and signaling through EGFR, lysophosphatidic acid and IGF. Proteins implicated in modulated pathways include MCL1, P38, ERBB2 and phosphorylation of RPS6 at S235/S236 and H2AX at S140. In addition, C-PAC modulated the DNA repair proteins RAD50 and RAD51.

In OE19 cells, AFG significantly modulates 49 proteins with 25 upregulated and 24 downregulated. As shown in Figure 7 and Supplementary Materials Table S4, HES1, P38, ERBB2 and phosphorylation of ERK1/2 at T202/Y204 and RB1 at S807/S811 contribute toward the top modulated process networks identified with linkages to NOTCH signaling, inflammation linked to neutrophil activation, amphotericin signaling and IL-2, as well as regulation of translation initiation and EMT. Top pathway maps significantly modulated by AFG included those involved in EGFR, IGF, ERK1/2, MTORC2 and vascular endothelial growth factor (VEGF) signaling as well as immune signaling through IL-2 and BAFF. Proteins significantly modulated by AFG driving these pathway maps include MTOR, RICTOR, HES1, MMP2, MCL1, P38 and phosphorylation of ERK1/2 at T202/Y204, RPS6 at S235/S236, H2AX at S140 and MTOR at S2448 (Figure 7).

RPPA analysis of lysates from JHAD1 and OE19 cells treated with the cranberry polyphenols C-PAC and AFG has provided additional insight into how these compounds result in cancer cell death. Overall, the AFG extract does not appear to be as potent as C-PAC at equivalent concentrations, and only 13% of proteins modulated are in common between the AFG and C-PAC treated JHAD1 cells illustrating that the majority of modulated proteins are unique to each treatment group despite similar effects on cancer linked processes. The data are similar for OE19 cells in that 25% of the total proteins significantly modulated are shared by C-PAC and AFG.

### 3.3. Cranberry Polyphenols Modulate P53-Linked, Inflammatory and Cell Cycle Proteins in BE and EAC Cells

**Figure 8:**
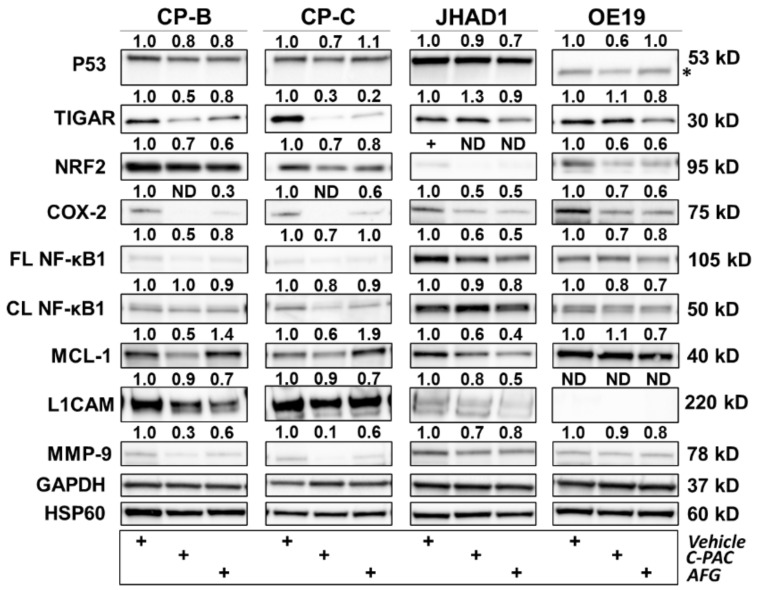
Polyphenols modulate several proteins related to P53, inflammation and metastasis in premalignant and EAC cell lines. CP-B, CP-C, JHAD1 and OE19 cells were treated with C-PAC (75 µg/mL, AFG (400 µg/mL) or vehicle and lysates isolated at 24 h following treatment. Western blotting was performed using commercially available antibodies to proteins of interest. Expression values were normalized to the appropriate loading control, GAPDH or HSP60, and fold change from vehicle was calculated using Imagelab. Bands with no detectable expression are denoted as ND. The asterisk (*) next to the P53 results in OE19 cells denote a faster migrating protein due to an insertion and duplication in the *TP53* coding region as described in the results and discussion. FL and CL denote full-length and cleaved proteins, respectively. The plus (+) sign denotes treatment group.

**Figure 9:**
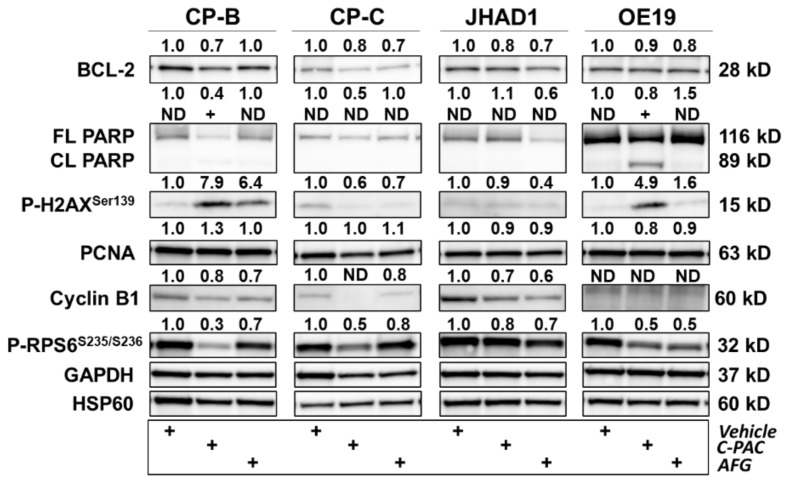
Polyphenols modulate several proteins related to DNA damage, cell cycle and growth arrest in premalignant and EAC cell lines. CP-B, CP-C, JHAD1 and OE19 cells were treated with C-PAC (75 µg/mL), AFG (400 µg/mL) or vehicle and lysates isolated at 24 h following treatment. Western blotting was performed using commercially available antibodies to proteins of interest. Expression values were normalized to the appropriate loading control, GAPDH or HSP60, and fold change from vehicle was calculated using Imagelab. Bands with no detectable expression are denoted as ND. FL and CL denote full-length and cleaved proteins, respectively. The plus (+) sign denotes treatment group.

**Figure 10:**
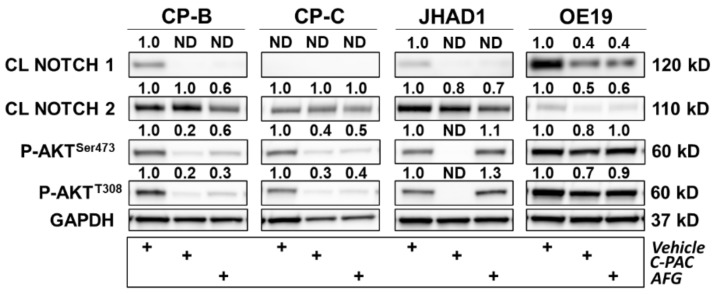
Polyphenols modulate several proteins involved in inflammation, DNA damage and cell cycle in premalignant and EAC cell lines. CP-B, CP-C, JHAD1 and OE19 cells were treated with C-PAC (75 µg/mL), AFG (400 µg/mL) or vehicle and lysates isolated at 24 h following treatment. Western blotting was performed using commercially available antibodies to proteins of interest. Expression values were normalized to GAPDH and fold change from vehicle was calculated using Imagelab. Bands with no detectable expression are denoted as ND. CL denotes a cleaved protein. The plus (+) sign denotes treatment group.

To further investigate and confirm the RPPA based results, we selected a panel of 18 proteins to perform Western blot analysis of BE and EAC cell lysates 24 h post polyphenol treatment, with results shown in Figure 8, Figure 9 and Figure 10. Analysis of proteins related to P53, inflammation and linked to metastasis are presented in Figure 6. Treatment with C-PAC decreased expression of mutant P53 in CP-B, CP-C, JHAD1 and OE19 cells, while AFG resulted in reduced mutant P53 only in CP-B and JHAD1 cells. It is important to note that point mutations in *TP53* are found in CP-B (c.524G > A), CP-C (c.742 C > T) and JHAD1 cells (c.797 G > A), while OE19 cells contain a duplication (c.929dup) and insertion (c.929_930ins1) resulting in a faster-migrating P53 protein (Figure 8, asterisk) [33]. TIGAR, a P53-inducible regulator of apoptosis and glycolysis [34], increased with C-PAC treatment in JHAD1 and OE19 cells, but decreased with AFG treatment in all cell lines. In contrast to EAC cells, C-PAC decreased TIGAR levels in CP-B and CP-C cells. In addition to its role in blocking glycolysis, TIGAR also plays a role in DNA repair through reductions in intracellular ROS levels [35]. Modulation of TIGAR in premalignant and EAC cells is consistent with our previous findings showing differential effects of C-PAC based on histopathology of the cell lines. Specifically, our earlier research revealed that C-PAC induces cell death through decreasing ROS in BE cells, while conversely increasing ROS leading to EAC cell death [22]. In terms of antioxidant responses [36], NRF2 levels decrease in CP-B, CP-C and OE19 cells with C-PAC and AFG treatment (Figure 8). NRF2 protein levels were barely detectable in JHAD1 cells. With respect to inflammation, C-PAC and AFG decrease COX-2 levels in all cell lines, with the most dramatic reductions observed in C-PAC treated CP-B and CP-C cells where levels are reduced to undetectable levels (Figure 8). Protein levels of the transcription factor NF-κB1, known to be activated by bile exposure and involved in activation of inflammatory signaling cascades in the esophagus, are decreased for both the full-length and cleaved forms in all cell lines with C-PAC and AFG treatment. Additionally, MCL-1, an outer mitochondrial membrane protein that regulates cell proliferation and apoptosis [37], is decreased in C-PAC treated CP-B, CP-C and JHAD1 cells, whereas, AFG treatment resulted in increased MCL-1 levels in premalignant BE cells, yet decreased expression in EAC cell lines. Consistent with the pathway enrichment results (Figure 4 and Figure 5), C-PAC and AFG decrease levels of the L1CAM cell adhesion molecule in JHAD1 cells, as well as in both premalignant cell lines. L1CAM was not detected in OE19 cells, which is interesting given that these cells are more bile-acid resistant and readily form xenografts in vivo [20] compared to JHAD1 cells. In terms of extracellular matrix remodeling, C-PAC and AFG decrease MMP-9 levels in all BE and EAC cell lines evaluated (Figure 8), supporting the enrichment results which revealed that C-PAC and AFG modulate EMT regulation.

We next investigated six proteins linked to DNA damage, cell cycle and growth arrest-induced cell death as presented in Figure 9. C-PAC and AFG decreased protein levels of BCL-2, an anti-apoptotic protein linked to DNA damage, in all cell lines evaluated. Based on the RPPA results, we also observed an increase in phospho-BAD^Ser122^, a BCL-2 interacting partner, for which the MAPK pathway regulates phosphorylation [38]. With respect to DNA damage, PARP cleavage was mainly observed with C-PAC treatment of OE19 cells, with lower magnitude effects for the BE progressor cell line, CP-B. Aligned with the latter results were concomitant increases in phospho-H2AX^Ser139^ for both CP-B and OE19 cell lines (Figure 9). AFG also induced DNA damage in these two cell lines but independent of PARP cleavage. We previously showed that C-PAC induces ROS, including hydrogen peroxide, in EAC cells, resulting in cell death [22]. Accumulation of ROS increases oxidative cellular stress levels which can lead to DNA, RNA, protein and lipid damage. RPPA analysis revealed that in OE19 cells, RAD50 and RAD51 were significantly upregulated following C-PAC treatment (Figure 6). Despite activation of DNA double strand repair pathways [39], OE19 cells succumb to C-PAC induced cell death, in part due to induction of DNA damage. With respect to DNA replication and cell cycle, C-PAC and AFG modestly decreased levels of PCNA in both JHAD1 and OE19. Based on Western blot results, PCNA levels increase in CP-B cells following C-PAC treatment, potentially due to activation of repair mechanisms in this BE-derived cell line.

Cyclin B1 levels were reduced following C-PAC and AFG treatment in CP-B, CP-C and JHAD1 cells, with levels not detected in OE19 cells even at baseline, consistent with our previous findings [20]. We next assessed the levels of phospho-RPS6^S235/S235^, a protein kinase substrate for which phosphorylation is stimulated by growth factors and mitogens and dephosphorylation is a sign of cellular growth arrest [40,41,42]. Both C-PAC and AFG treatment decrease levels of phospho-RPS6^S235/235^ in BE and EAC cell lines (Figure 9) and these results are consistent with the decrease noted in cellular viability observed in Figure 1, Figure 2 and Figure 3.

The last panel of four proteins evaluated based on the RPPA results are related to NOTCH signaling and are depicted in Figure 10. Bioinformatic analysis revealed the NOTCH signaling process network was among the top ten process networks significantly modulated in JHAD1 and OE19 cells treated with either C-PAC or AFG (Figure 4, Figure 5, Figure 6 and Figure 7). Western blot results further support that C-PAC and AFG decreased levels of the NOTCH1 intracellular domain in CP-B, JHAD1 and OE19 cells; levels were undetectable in CP-C cells. In premalignant CP-B cells, AFG, but not C-PAC, reduced levels of NOTCH2 intracellular domain. C-PAC and AFG reduced levels of NOTCH2 intracellular domain in JHAD1 and OE19 cells.

As it relates to the RPPA platform, care must be taken when assessing the levels of protein in which the antibody recognizes multiple forms, i.e., full-length and cleaved. For example, in our RPPA dataset, C-PAC increases levels of NOTCH1 in JHAD1 cells (Figure 4), which is counterintuitive given cancer cell death induction; however, Western blot analysis revealed that C-PAC specifically decreases the intracellular cleaved form of NOTCH1 (Figure 10). Given that increased NOTCH signaling is linked to phosphorylation of the PI3K/AKT pathway [43,44], we next assessed levels of phosphorylated AKT. C-PAC and AFG decreased levels of phospho-AKT^Ser473^ and phospho-AKT^T308^ in CP-B, CP-C and OE19 cells. C-PAC decreased levels of both phospho-AKT^Ser473^ and phospho-AKT^T308^ in JHAD1 cells, which is consistent with our previously published results [20]. By and large, AFG did not reduce phosphorylation of AKT in JHAD1 cells and only had a minor impact on OE19 EAC cells highlighting that, although the two fractions work through common signaling nodes, there are examples of divergence among select molecules.

## 4. Discussion

Considering the poor 5-year survival statistics and rising incidence of EAC, there is a critical need for improving targeted efforts for prevention and treatment of this deadly malignancy. We and others have previously shown that cranberry extracts have cancer inhibitory properties in numerous in vitro studies utilizing an array of diverse cancer cell lines [11,12,13,14,15]. Additionally, a few in vivo studies, largely centered around carcinogen-induced and xenograft models, have supported cranberries’ cancer inhibitory potential [11,16,17,18]. Few clinical studies targeting cancer or premalignancy have been conducted with cranberry or cranberry derivatives to date; however, the limited research does support antibacterial activity, favorable effects on the microbiome, and one study reported a 22.5% drop in PSA levels following a 30 day intervention [45,46,47,48,49,50].

As an extension of completed research, we conducted studies using multiple premalignant BE and EAC cell lines to further assess mechanisms associated with the cancer inhibitory potential of two cranberry extracts, one rich in proanthocyanidins and the other a combination of anthocyanins, flavonoids and glycosides. The first part of our study assessed the ability of the cranberry polyphenols, C-PAC or AFG, to inhibit the viability of human derived esophageal cell lines across a continuum ranging from premalignant BE derived lines to cell lines originating from esophageal adenocarcinoma patients. Previous in vitro studies have shown that cranberry constituents exhibit cancer inhibitory activity toward more than 50 cancer cell lines from a range of targets, as previously reviewed [11]. Specifically, either anthocyanins or flavonoid-glycoside rich extracts decrease the viability of several cancer cell lines, including those derived from colon, breast, glioblastoma, liver, melanoma and prostate [51,52,53,54]. To our knowledge, this study is the first to evaluate anthocyanins, flavonoids and glycosides as one extract. Overall, C-PAC is approximately eight-fold more potent at killing CP-B and CP-C cells than AFG, even at high AFG concentrations (Figure 2 and Figure 3). Interestingly, phytochemical profiling of the cranberry as summarized by Blumberg et al. reports that proanthocyanidins comprise approximately 25% of the cranberry, with other constituents making up the remaining 75%, which includes the anthocyanins, flavonoids and glycosides amongst other bioactive components [55]. The latter raises the question of whether the fact that C-PACs enhanced cancer inhibitory potential and at lower concentrations compared to AFG may simply parallel the relative quantities of these fractions, which occur naturally in the plant. In turn, constituents including AFG may require higher concentrations to exert the same effects, as they represent a larger fraction of the whole cranberry. Consistent with our data (Figure 1, Figure 2 and Figure 3), treatment with a methanol soluble flavonoid enriched extract (Fr6) in the range of 21–234 µg/mL was necessary to reach an IC_50_ in breast, colon and prostate cell lines [52]. Furthermore, in an U87 glioblastoma xenograft model in female NCR NU/NU mice, the Fr6 fraction at 250 mg/kg was able to significantly delay tumor growth at a rate equivalent to 100 mg/kg of proanthocyanidins [51]. Importantly, levels of C-PAC and AFG that demonstrate cancer inhibitory activity are behaviorally achievable through consumption of fresh cranberries, a cranberry juice cocktail or sweetened dried cranberries [19,48,53,55].

RPPA results support that C-PAC and AFG treatment have rather pleiotropic effects on diverse cellular processes, ultimately resulting in EAC cell death. C-PAC and AFG significantly modulated the signal transduction process network NOTCH signaling. Increased NOTCH signaling is commonly observed in cancer and chemoresistance [56], with polyphenols including quercetin and resveratrol reportedly reducing NOTCH intracellular domain protein levels [57,58]. Interestingly, polyphenols including picatannol, apigenin, chrysin and genistein upregulate NOTCH signaling to elicit anti-cancer effects via inhibition of cellular proliferation and migration [59,60,61]. NOTCH signaling is increased in progression from BE to EAC [62,63,64,65] and decreased NOTCH signaling inhibits EAC xenografts [66]. The ability of cranberry polyphenols to downregulate the intracellular domains of NOTCH1 and NOTCH2 is promising for targeting NOTCH-linked progression of BE to EAC (Figure 10). Kunze et al. recently showed that *NOTCH2* and *NOTCH3* were upregulated with progression to EAC in human tissues and expression of the intracellular domain of NOTCH2 led to increased dysplasia and decreased survival rates in the *L2-Il1B* mouse model of BE [65]. Our data show that the NOTCH signaling pathway is significantly modulated by C-PAC and AFG and that the canonical NOTCH target and transcriptional repressor HES1 [67] are downregulated by C-PAC and AFG in JHAD1 cells and by AFG in OE19 cells. C-PAC and AFG impact cell cycle processes in both JHAD1 and OE19 cells, with G1-S and G2-M highlighted in the process networks. The results for Cyclin B1 are consistent with our previous findings reporting that C-PAC induces cell cycle arrest at G2-M, elicits an S-Phase delay and largely induces caspase-independent cell death, which is also consistent with the enrichment analysis herein [20].

C-PAC and AFG similarly modulate inflammation and immune responses. The immunosuppressive tumor microenvironment in EAC, not only results in immunotherapy being relatively ineffective but also allows tumors to evade cell death and continue to aggressively grow [68,69]. Immune cells that surround BE and EAC, including myeloid-derived suppressor cells, T-regulatory cells and Th17 cells, secrete proinflammatory cytokines including IL-6, IL-19, TNFα and TGF-β, resulting in a tumor permissive environment that promotes tumor cell survival, proliferation and metastasis [70,71,72]. In alignment with the anti-inflammatory capacity of cranberry polyphenols [73], C-PAC and AFG modulated several anti-inflammatory process networks including those linked to IL-2, IL-6, IL-10 signaling and neutrophil activation as well as immune response pathway maps related to interferon, IL-2, IL-3, IL-4, IL6 and BAFF signaling. One additional pathway map to highlight from the RPPA analysis is that of oxidative stress due to reactive oxygen species (ROS)-induced cellular signaling in JHAD1 cells. These results are consistent with data we previously published [22] showing that C-PAC induces ROS in both JHAD1 and OE19 cells leading to cell death, but more so in JHAD1 cells, which aligns with oxidative stress being the most modulated pathway by C-PAC in JHAD1 cells. Additionally, oxidative stress due to the ROS-induced cellular signaling pathway map is the 13th-most significantly modulated in OE19 cells, further highlighting its central role in cell death induction in both cell lines (see Table S3). Protein level results following C-PAC, and to a lesser extent AFG treatment, also align with oxidative-linked changes as evidenced by increased levels of the DNA damage marker phospho-H2AX^Ser139^ in OE19 and CP-B cell lines. OE19 is a phenotypically aggressive EAC cell line and, although CP-B is a BE derived cell line, the patient from which the line was developed progressed to EAC, supporting the notion that this BE line was rewired prior to pathologic progression to EAC. This concept aligns with recent research by Fitzgerald et al. which identified *TP53* mutational events among EAC progressors prior to the onset of high-risk pathology [74]. Our results support that both C-PAC and AFG possess cancer inhibitory properties, but the precise molecules modulated may reflect cell line diversity and patient heterogeneity.

Given that *TP53* is the best-known and most frequent mutation involved in EAC development [5,75,76], the ability of cranberry polyphenols to target mutant P53 and key signal transduction pathways implicated in EAC progression is promising and warrants further investigation in early phase clinical trials. Our results show that cranberry polyphenols reduce P53 levels by 10–40%, presumably reflecting reduced levels of the mutated form of the gene. This phenomenon is encouraging to note in BE-derived cell lines which may reflect sensitivity of premalignant esophageal cells to *TP53* reprogramming by polyphenols. In alignment with our results, others have shown that polyphenols including curcumin, resveratrol, epigallocatechin-3-gallate, as well as other black and green tea derivatives, alter *TP53* signaling pathways, leading to cell death in a variety of cancer cell lines [11].

Matrix metalloproteases, including MMP-9, are linked to progression in many cancers and are responsible for the remodeling of the extracellular matrix, resulting in tumor invasion and metastasis [77]. Increased levels of MMP-9 are observed in tissues from patients with dysplastic BE and EAC, but not those with esophagitis or metaplastic BE [78,79]. Furthermore, MMP-9 expression is inversely correlated with protein levels of the phase II detoxification glutathione-s-transferase pi (GSTP1), with increased levels of GSTP1 in normal patients and those with esophagitis [79]. We recently showed that C-PAC increases levels of the phase 2 detoxification enzyme glutathione-s-transferase theta 2 (GSTT2) and protects against acidified bile acid induced cell death in patient-derived primary normal esophageal cells [80]. Thus, the ability of cranberry constituents to favorably impact esophageal cells, both primary and immortalized cell cultures, across a range of histopathology lends support for the investigation of cranberry constituents in cohorts at increased risk for progression to EAC, such as BE patients. Consistent with decreased MMP-9 levels, C-PAC and AFG decreased expression of L1CAM in JHAD1 cells, a neuronal adhesion protein linked to metastasis and chemoresistance, and is overexpressed in many solid tumor cancers including esophageal squamous cell carcinoma and gastric cancer [81,82,83]. AFG modulated similar process networks as C-PAC in JHAD1 cells including cell cycle kinetics, but also different examples including downregulation of epithelial to mesenchymal transition (EMT) and proinflammatory signaling cascades involving IL-2 and IL-4. Both C-PAC and AFG modulated ERBB family signaling in JHAD1 and OE19 cells. ERBB family members including ERBB1/EGFR and ERBB2/HER2, are receptor tyrosine kinases that are often aberrantly activated leading to cancer cell migration, EMT, antitumor immunity and cancer cell survival [84,85]. Upregulation of both ERBB1/EGFR and ERBB2/HER2 is observed in progression from BE to EAC and increased surface expression has been used for in vivo detection using fluorescent peptides targeting these two proteins [86,87,88,89,90].

The ability of C-PAC and AFG to decrease NRF2 in BE and EAC cell lines is intriguing as the NRF2/KEAP1 pathway is upregulated in BE and EAC based on recent COSMIC database analysis [91]. Polyphenols including luteolin, apigenin and chrysin inhibit NRF2 in multiple cancer cell lines resulting in inhibition of multidrug-resistant drug transporters, increased sensitivity to chemotherapeutic drugs and ROS-induced cell death [92]. Finally, there is preliminary data suggesting that brusatol, a natural compound and NRF2 inhibitor [93], selectively kills EAC cells versus normal or premalignant metaplastic BE cells and increases sensitivity of EAC cells to the chemotherapeutic agent cisplatin via increased ROS [94]. Therefore, the ability of C-PAC and AFG to downregulate NRF2 protein levels in BE and EAC cells is promising.

## 5. Conclusions

Study results support that cranberry polyphenols inhibit multiple cancer associated processes in EAC and BE cell lines, contributing significantly to reduced cellular viability and induction of cell death. We previously utilized EAC cell lines and an EAC xenograft model to investigate the cancer inhibitory effects of C-PAC via the PI3K, MAPK, AKT and MTOR signaling cascades as well as cell death induction through apoptosis, autophagy and necrosis [20]. However, the AFG fraction has not previously been investigated. Our current research revealed that C-PAC and AFG cranberry extracts mitigate key networks dysregulated in EAC progression including NOTCH, NRF2, immune, EMT and TP53 signaling.

The capacity to simultaneously assess levels of over 300 proteins via the RPPA platform greatly aided our ability to dissect pathways impacted by cranberry polyphenols, as well as to identify specific molecules as potential new targets (i.e., P53, NOTCH1, etc.). Moreover, *TP53* mutations can occur early during BE with increased events noted with pathologic progression and therefore may serve as a logical target for EAC inhibition. EMT-linked alterations were also mitigated by cranberry polyphenols, particularly C-PAC, warranting additional research to evaluate whether C-PAC pretreatment diminishes therapeutic resistance in EAC.

Finally, future directions will include investigating the mechanisms by which cranberry polyphenol treatment results in premalignant and cancer cell death following exposure to acidified bile, the strongest consistent risk factor for BE development and EAC progression. In addition, early phase clinical trials evaluating cranberry constituents in patients with gastroesophageal reflux disease (GERD) or Barrett’s precursor lesions may prove beneficial considering mounting preclinical evidence that cranberries mitigate the major molecular drivers and risk factors associated with EAC progression. GERD and BE are associated with increased levels of pathogenic bacteria, whereas sweetened dried cranberries reportedly decrease pathogenic bacteria in healthy subjects [49]. The latter study is promising in two ways; its positive effects were noted after just two weeks and the level of cranberries required to illicit positive effects was a single ½ cup serving of dried cranberries each day. A recent 8-week double-blind randomized placebo-controlled trial of C-PAC juice found that 44 mg PAC equivalents twice daily inhibited *Helicobacter pylori* infection [46]. C-PACs have also been reported to possess anti-adhesion effects toward *Escherichia coli*, *Streptococcus mutans*, and *Candida albicans* which are implicated in oral biofilms [8,95,96,97,98,99]. However, to our knowledge clinical trials delivering cranberry-based products or extracts have not been conducted in patients at increased risk for esophageal cancer.

## Data Availability

The data that support the study findings are available from the corresponding author upon reasonable request.

## Supplementary Materials

The following are available online at https://www.mdpi.com/article/10.3390/nu14050969/s1, Table S1: Metacore analysis for JHAD1 cells and C-PAC treatment, Table S2: Metacore analysis for JHAD1 cells and AFG treatment, Table S3: Metacore analysis for OE19 cells and C-PAC treatment, Table S4: Metacore analysis for OE19 cells and AFG treatment.
